# Recovery and resiliency of skin microbial communities on the southern leopard frog (*Lithobates sphenocephalus*) following two biotic disturbances

**DOI:** 10.1186/s42523-020-00053-5

**Published:** 2020-09-22

**Authors:** Denita M. Weeks, Matthew J. Parris, Shawn P. Brown

**Affiliations:** 1 Department of Biology, Grand Junction, Colorado Mesa University, Grand Junction, CO 81501 USA; 2 Department of Biological Sciences, The University of Memphis, Memphis, TN 38152 USA; 3 Center for Biodiversity Research, The University of Memphis, Memphis, TN 38152 USA

**Keywords:** Biopesticide, Microbiome, Amphibian, Disturbance, *Bd*, *Batrachochytrium dendrobatidis*

## Abstract

**Background:**

Microorganisms have intimate functional relationships with invertebrate and vertebrate taxa, with the potential to drastically impact health outcomes. Perturbations that affect microbial communities residing on animals can lead to dysbiosis, a change in the functional relationship, often associated with disease. *Batrachochytrium dendrobatidis* (*Bd*), a fungal pathogen of amphibians, has been responsible for catastrophic amphibian population declines around the globe. Amphibians harbor a diverse cutaneous microbiome, including some members which are known to be antagonistic to *Bd* (anti*-Bd*)*.* Anti-*Bd* microorganisms facilitate the ability of some frog populations to persist in the presence of *Bd,* where other populations that lack anti-*Bd* microorganisms have declined. Research suggests disease-antagonistic properties of the microbiome may be a function of microbial community interactions, rather than individual bacterial species. Conservation efforts have identified amphibian-associated bacteria that exhibit anti-fungal properties for use as ‘probiotics’ on susceptible amphibian populations. Probiotic application, usually with a single bacterial species, may benefit from a greater understanding of amphibian species-specific microbiome responses to disturbances (e.g. dysbiosis vs. recovery). We assessed microbiome responses to two microbial disturbance events over multiple time points.

**Results:**

Exposing *Lithobates sphenocephalus* (southern leopard frog) adults to the biopesticidal bacteria *Bacillus thuringiensis,* followed by exposure to the fungal pathogen *Bd,* did not have long term impacts on the microbiome. After initial shifts, microbial communities recovered and returned to a state that resembled pre-disturbance.

**Conclusions:**

Our results indicate microbial communities on *L. sphenocephalus* are robust and resistant to permanent shifts from some disturbances. This resiliency of microbial communities may explain why *L. sphenocephalus* is not experiencing the population declines from *Bd* that impacts many other species*.* Conservation efforts may benefit from studies outlining amphibian species-specific microbiome responses to disturbances (e.g. dysbiosis vs. recovery). If microbial communities on a threatened amphibian species are unlikely to recover following a disturbance, additional measures may be implemented to ameliorate the impacts of physical and chemical stressors on host-associated microbial communities.

## Background

Microorganisms can play an integral role in the health, development, and host fitness of vertebrates through numerous pathways including immunocompetence and pathogen defense [1]. For instance, the absence of some cutaneous bacterial taxa on amphibians has been associated with higher infection susceptibility and mortality rates from the fungal pathogen *Batrachochytrium dendrobatidis* (*Bd*) [2]. *Bd* is the causal agent of chytridiomycosis, which elicits reduced physiological processes in symptomatic amphibians including electrolyte transport, immune function, and epidermal cell association that leads to cell death [3–6]. Amphibian populations that have not experienced major declines when *Bd* is present have been associated with the number of individuals carrying anti*-Bd* bacteria [2]. The *Bd* antagonistic properties found in some cutaneous bacteria associated with amphibians are often manifested via antifungal secondary metabolites that inhibit *Bd* growth and/or promote *Bd* cell lysis [7–10] in vitro. Yet, the protective function of the microbial community, or microbiome, may depend on community interactions. Loudon et al. [11] found that two bacterial species known to inhibit *Bd* growth exhibited greater anti-*Bd* activity in vitro when co-cultured. This suggests that some disease mitigation properties of bacteria may be synergistic and only occur when both are present, underscoring the importance of community interactions in these processes. These community interactions can be disrupted via disturbances (e.g., habitat alteration, pollution, invasive species, parasites), potentially altering the protective properties of the microbiome reviewed in [12]. As amphibian populations are exposed to an increasing frequency of disturbances, conservation efforts will be complimented by a better understanding of microbiome responses to disturbances and how this may differ among amphibian species [12, 13]. Additionally, research or conservation-related practices can disrupt the microbiome. Animal handling in the wild, even if brief, to equip amphibians with passive integrated transponders can temporarily disrupt microbiomes [14]. Also, individuals housed for reintroduction in captive breeding programs experience a substantial loss in cutaneous microbiome diversity, including crucial *Bd*-inhibitory microbes [15]. However, a probiotic treatment with the anti-*Bd* bacterium *Janthinobacterium lividum* has been demonstrated to successfully restore protective function against *Bd* [15]. Understanding events that lead to dysbiosis, a change in the functional relationship of the microbial community with the host, and which exhibit transient effects on the microbiome may assist in developing mitigation strategies for imperiled populations.

Amphibians often maintain a core cutaneous microbial community (here defined as taxa found on at least 90% of all individuals within a population) which may initially colonize from environmental reservoirs, such as soil and water [8, 12, 16, 17] and remain important members of the community. Some of these core taxa are thought to have important roles in host health and recovery following disturbance events [18]. However, core taxa membership is driven largely by the amphibian species rather than environmental attributes, suggesting that assembly of core taxa may be more deterministic rather than haphazardly acquired [16, 19, 20]. While some core cutaneous taxa have been cultured and their anti-fungal activities verified [21], surveys of the entire microbial community, including non-culturable members, can provide additional insight into how community biodiversity might shift and/or recover following perturbation events. Perturbation events may shift the functional relationship between the host and the microbiome, causing dysbiosis that leads to decreased protection from potential pathogens [22]. If and how the microbiome recovers following a perturbation is essential information for conservation efforts on endangered taxa when prioritizing management strategies, determining at-risk populations, and reintroducing animals from captive breeding programs [8, 11].

While dysbiosis in the microbiome may cause increased infections from pathogens, pathogen colonization can also lead to dysbiosis. Jani & Briggs [23] found low variability in the bacterial communities within three populations of *Rana sierra* (Sierra Nevada yellow-legged frog) until a *Bd* epizootic event occurred in one population. This epizootic event led to drastic changes in bacterial β-diversity. Subsequent laboratory experiments indicated that *Bd* infection induced dysbiosis by increasing the abundance of some microbial taxa while decreasing others. This may alter the protective relationship between the host and microbiota that existed prior to the *Bd* disturbance. Thus, *Bd-*induced disturbances may contribute to symptoms of chytridiomycosis in diseased individuals via reduced protective processes. Jani & Briggs [17] further found that the degree of *Bd-*induced shifts in the bacterial communities may rely on *Bd* infection intensity. When they assessed communities 28 days after *Bd* exposure, they reported changes in relative abundance of some bacterial lineages correlated with *Bd* loads. However, it is unknown if or how *Bd* elicits microbiota dysbiosis in other amphibian species, or if these changes are transient.

Disturbances to the microbiome can also be caused by xenobiotics, including pesticides. Exposure to a glyphosate-based herbicide induced mortality and significantly altered the cutaneous bacterial community in surviving juvenile *Acris blanchardi* (Blanchard’s cricket frog) [24]. Rumschlag and Rohr [25] examined associations between pesticide use and *Bd* infection prevalence and found that herbicide exposure is associated with increased risk of *Bd* infections and associated mortality later in life, which may be linked to decreased immunocompetence [24, 26]. While research has confirmed that chemical pesticides have the capacity to alter microbial communities living on amphibian skin, the potential effects of microbial biocides have yet to be investigated. Biocides, some of which include live microbes, exhibit anti-pest properties. As biotic agents, microbial biocides may compete with resident bacteria for space and nutrients or exhibit niche complementarity. Over 90% of commercially available biocide formulations are derived from the bacteria *Bacillus thuringiensis* [27]. This species produces a crystal protein (Cry toxin) that has insecticidal action on specific insect groups when ingested. The Cry toxins must encounter specific conditions to become toxic, including an alkaline gut pH found in the gut of an insect, and specific receptors on the gut epithelia. Through this multistep process, the Cry toxin causes gut membrane perforation and septicemia in the insect [28]. These biocides often impact aquatic environments, either through direct aquatic applications or runoff from terrestrial applications (e.g. agricultural fields). *B. thuringiensis* spores can persist and be viable for at least 13 years in soils after an application event [29] and has been found in rivers and public waters weeks after aerial applications [30]. While *B. thuringiensis* is found naturally and is abundant in soils across the globe [31], the widespread application of *B. thuringiensis* formulations may increase the abundance of this species in the environment. It is unknown, however, if *Bacillus thuringiensis* biocides have impacts on the microbiome of amphibians*.*

Few studies have explored the dynamic changes that occur among cutaneous amphibian microbiota. Evidence suggests that environmental microbial reservoirs are important in the development and regulation of the core microbiome. Loudon et al. [11] investigated if a natural soil reservoir was necessary for *Plethodon cinereus* salamanders to maintain bacterial communities in captivity. While the environmental reservoir was shown to have an impact, results also suggested that many bacteria persisted on the amphibian host, even when the amphibians were housed with sterile soil. These host-associated taxa may initially colonize (founders) from the environmental reservoir and remain important members of the core community, with the ability to be resilient and recover from disturbances. To our knowledge, our study is the first to investigate how the cutaneous amphibian core microbiome, as well as the whole community, responds to disturbances from a microbial biocide and a fungal disease of the skin over time. It is also the first to investigate the colonization potential of the common biocide bacterium *Bacillus thuringiensis.*

To investigate this, we used a locally-reared population of *Lithobates sphenocephalus* (southern leopard frog) as a model host. The culturable microflora was previously characterized from this population [21]. This species is susceptible to *Bd* infection but is moderately resistant to disease effects [32]. We hypothesized that (1) exposure to *Bacillus thuringiensis subsp. kurstaki* (*Btk*) from a biopesticide would increase *Btk* abundance in the community and (2) would elicit shifts in the microbiome. Based on work by Jani & Briggs [23], we also hypothesized that (3) exposure to *Bd* would further disrupt the cutaneous microbial community to a dysbiotic or alternative stable state.

## Results

**Fig. 1:**
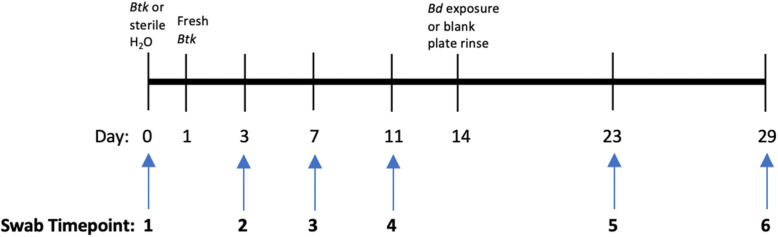
Timeline for bacteria (*Btk*) exposures and *Bd* exposures. Timepoints for swabs are also included

*Lithobates sphenocephalus* adults were assigned into four treatment groups: N (negative control; *n* = 6), *Btk* (*B. thuringiensis kurstaki* exposure; *n* = 8)*, Bd* (*B. dendrobatidis* exposure; *n* = 7)*,* BB (*Btk* + *Bd; n* = 10). Swab samples were taken at 6 timepoints over the course of the experiment (*n* = 186 swabs). These include: Day 0 (Timepoint 1), Day 3 (Timepoint 2), Day 7 (Timepoint 3), Day 11 (Timepoint 4), Day 23 (Timepoint 5), and Day 29 (Timepoint 6) (Fig. 1).

### Sequence information

Initial Illumina MiSeq metabarcoding yielded ~ 13.2 million paired sequences from 153 samples (all amplified PCR products from individuals across all time points and treatments). Average sequences per sample were ~ 38,500. (±1945 s.e.). After sequence quality control and elimination of operational taxonomic units (OTUs) that were found at a count of less than 10 globally, ~ 5.8 million sequences were retained and demarcated into 2343 OTUs. To determine which OTU corresponded to the strain of *Btk* used in exposures, a representative sequence from *Btk* was obtained (see below) and was found to be a perfect match (100% identity) to our obtained OTU72 (hereafter referred to as *Btk* 72).

### *Bd* infection

In the group treated with *Bd* only, there was 100% infection prevalence with *Bd* genetic equivalents during timepoint 5 and timepoint 6 of 1681.9 (± 649.3 s.e.) and 6581.3 (± 2311.9 s.e.), respectively. However, in the BB group, there was a 60% infection prevalence. The *Bd* genomic equivalents for individuals in this group during timepoint 5 and timepoint 6 were 877.8 (± 257.1 s.e.) and 2672.6 (± 666.8 s.e.), respectively. There was no association found between *Btk* 72 and *Bd* genomic equivalents at timepoint 5 (t = 2.61, *P* = 0.356) or at timepoint 6 (t = 1.38, *P* = 0.199) based on regression analyses.

### Diversity indices

**Fig. 2:**
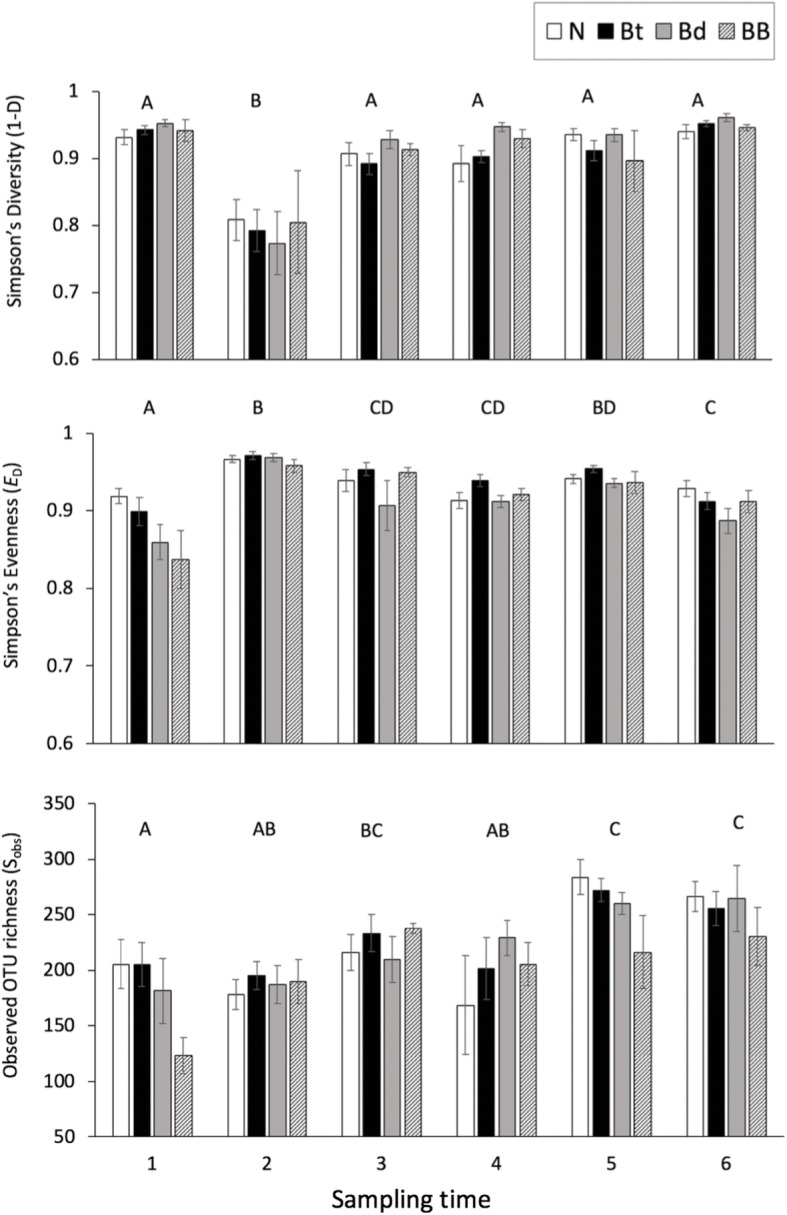
Alpha diversity of cutaneous microbial communities across treatment groups and sampling time (reference Fig. 1). **a** Simpson’s diversity differed among sampling time but treatment did not have an impact. **b** Simpson’s evenness (*E*_D_) differed over treatments and time. **c** Observed OTU richness (S_obs_) differed across sampling time but not treatments. The legend is applicable to all biodiversity figures. Letters denote significant differences among sampling times. Repeated measures ANOVA statistics are listed in Table 1. Values are the mean (± s.e.) of the biodiversity statistics calculated by mothur

OTU diversity (1-D; the complement of Simpson’s diversity) differed with sampling timepoints (F_5,103.84_ = 21.22, *P* < 0.0001) with large effect but not with treatments or their interactions (Table 1). Diversity decreased precipitously from timepoint 1 and timepoint 2 (following the first *Btk* bath; Fig. 2) but recovers quickly and remains consistent for remaining sampling timepoints.

**Table 1:** Results of repeated measures ANOVAs on diversity estimators across treatment, time and their interaction. F statistics are included with degrees of freedom (F_df, dfDen_) where dfDen is the denominator df based on Kenward-Roger first order approximations with Kacker-Harville corrections. Where significant, partial *η*^2^ effect sizes are presented parenthetically

Diversity Estimators	F-statistic	***p***-value
**Diversity (1-D)**		
Treatment	F_3,20.05_ = 0.62	0.6078
Time	**F**_**5,103.84**_ **= 21.22**	**< 0.0001 (0.460)**
Treatment x Time	F_15,102.87_ = 0.45	0.9591
**Evenness (E**_**D**_**)**		
Treatment	**F**_**3,24.13**_ **= 5.88**	**0.0037 (0.122)**
Time	**F**_**5,107.50**_ **= 19.22**	**< 0.0001 (0.436)**
Treatment x Time	F_15,106.67_ = 1.32	0.2039
**Richness (S**_**obs**_**)**		
Treatment	F_3,114.6_ = 2.43	0.0688
Time	**F**_**5,102.0**_ **= 11.05**	**< 0.0001 (0.269)**
Treatment x Time	F_15,101.2_ = 1.12	0.3462

(Tukey’s HSD). Community evenness (E_d_) shifts with both treatment (*P* = 0.0037) and time (*P* < 0.0001), but the interaction was never significant. Evenness increased over time indicating a decrease in taxa dominance (Fig. 2). Treatment effects on evenness indicate that frogs treated with *Bd* (the Bd group and the BB group were most similar) have similar evenness and both groups treated with only bacteria did not differ from the negative control (Fig. 2). Observed relative OTU richness (S_obs_) did not differ with treatment but increased with time (*P* < 0.0001; Table 1; Fig. 2).

### Community dynamics

**Fig. 3:**
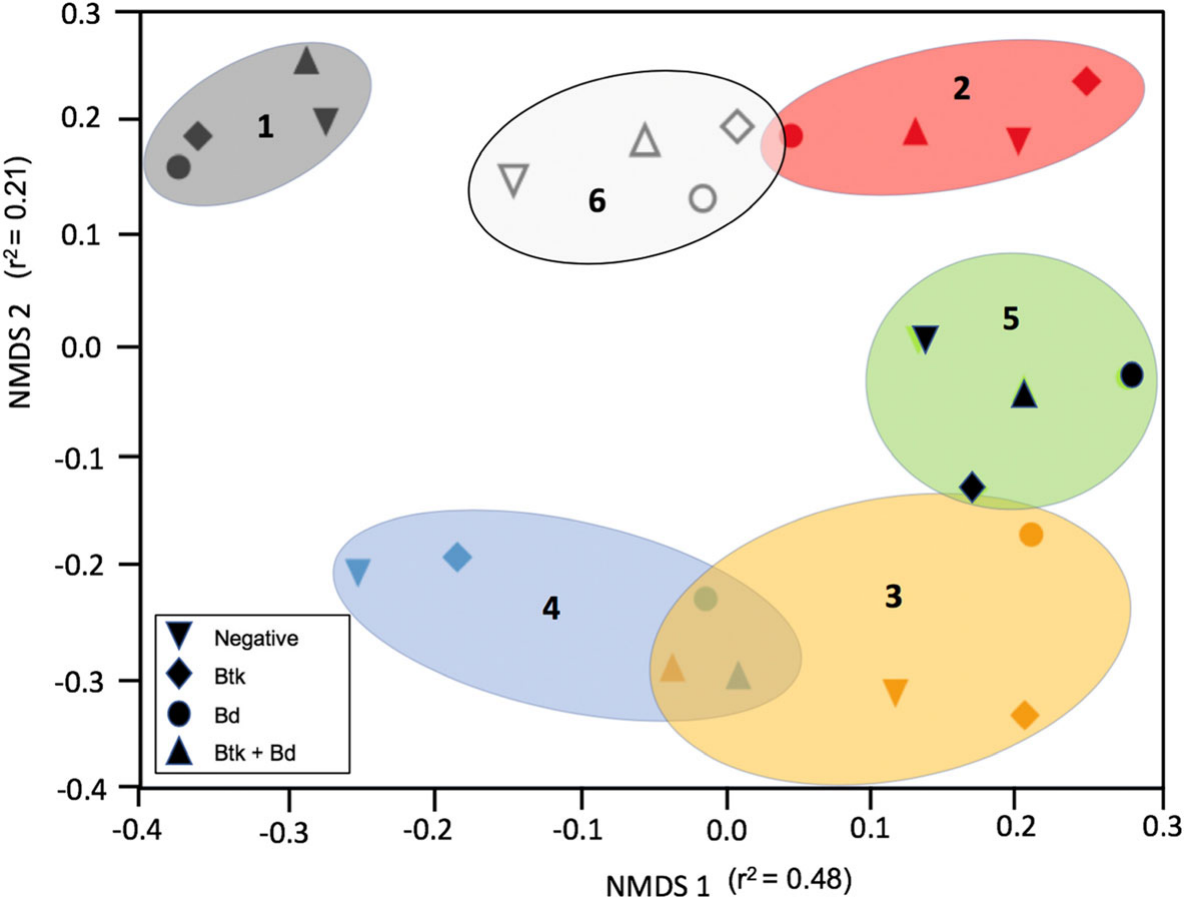
Within-treatment variation in microbial communities over sampling time. Non-metric Multidimensional Scaling (NMDS) ordination using Bray-Curtis dissimilarity distances. Each symbol represents an average of the Bray-Curtis value for each treatment group. Ellipses group sampling time together and include a label to indicate the sampling period. 4D stress = 0.15

Cutaneous microbial community structure shifts with treatment, time, and treatment x time interactions based on PERMANOVA results (Table 2) on Bray-Curtis dissimilarity values, but time is the strongest driver of community shifts (R^2^ = 0.378) and treatment and time x treatment interactions suggest a more minor role in community changes (R^2^ = 0.017 and R^2^ = 0.085 respectively). The NMDS ordination (Fig. 3; 4D stress = 0.15) and pairwise PERMANOVA treatment comparisons (Table 2) suggests that observed differences in community structure were greater among timepoints than among experimental treatments. Examination of changes in community structure within individual frogs in multidimensional ordination space over time, with treatment, and their interactions was conducted on AWOrD (Axes Weighted Ordination Distance) values with a two-way ANOVA model, this indicated an overall effect (F_19,124_ = 3.482, *P* < 0.0001) with time and treatment effects also being significant, but not the interaction (Table 3). This indicates that the weighted distance in ordination space is lower (communities more similar) between timepoint 1 and timepoint 6 than ordination distances between timepoint 1 and other sampling timepoints on individual frogs. So, while communities changed with time, they recovered quickly and became more similar to pre-treatment communities by the end of our experimental framework but not identical (Tables 2 and 3, Fig. 2).

**Table 2:** Results from PERMANOVA analysis of bacterial communities across treatment, time, and their interaction as well as residuals. Connecting letters reports are presented using post-hoc pairwise PERMANOVAs where sampling events (1–6) that have different letters differ in community structure (*P* < 0.05)

	Pseudo-F_**df**_		***p***-value		***R***^***2***^	
Treatment	F_3,148_ = 1.37		0.001		0.017	
Time	F_5,148_ = 18.18		0.001		0.378	
Treatment x Time	F_15,148_ = 1.36		0.016		0.085	
Residuals					0.520	
**Treatment**	**1**	**2**	**3**	**4**	**5**	**6**
N	A	B	C	CD	E	F
Btk	A	B	C	D	E	F
Bd	A	B	C	CD	DE	F
BB	A	B	C	CD	E	AF

**Table 3:** Results of pairwise ANOVA tests of Axis Weighted Ordination Distance (AWOrD) values across treatment (N, Btk, Bd, BB), time contrasts (T1 vs. T2, T1 vs. T3, T1 vs. T4, T1 vs. T5, T1 vs. T6) and their interactions for the same individual frog. Presented are sum of squares, F-statistics, P-value, and presented in superscripts are the results of post-hoc Tukey HSD (connecting letters) and AWOrD means for each grouping

Test	SS	F_**df**_	***P***-value	Tukey HSD and Means
Treatment	0.0771	F_3,105_ = 3.244	0.025	N^AB (0.279)^, Btk^A (0.321)^, Bd^A (0.308)^, BB^B (0.255)^
Time	0.3196	F_4,105_ = 10.077	< 0.001	1-2^AB (0.293)^, 1–3^A (0.337)^, 1-4^BC (0.256)^, 1-5^A (0.354)^, 1-6^C (0.214)^
Treatment x Time	0.0874	F_12,105_ = 0.918	0.532	

**Fig. 4:**
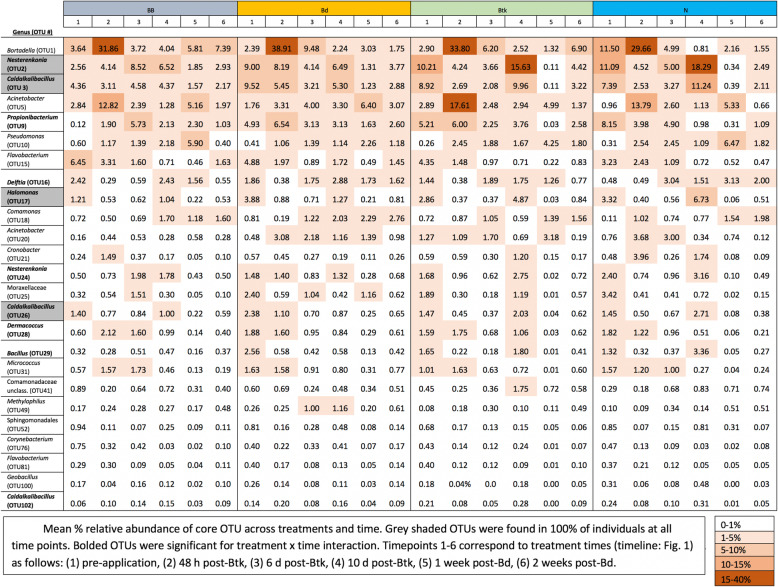
Mean % relative abundance of core OTU across treatments and time. Grey shaded OTUs were found in 100% of individuals at all time points. Bolded OTUs were significant for treatment x time interaction. Timepoints 1–6 correspond to treatment times (timeline: Fig. 1) as follows: (1) pre-application, (2) 48 h post-Btk, (3) 6 d post-Btk, (4) 10 d post-Btk, (5) 1 week post-Bd, (6) 2 weeks post-Bd

**Fig. 5:**
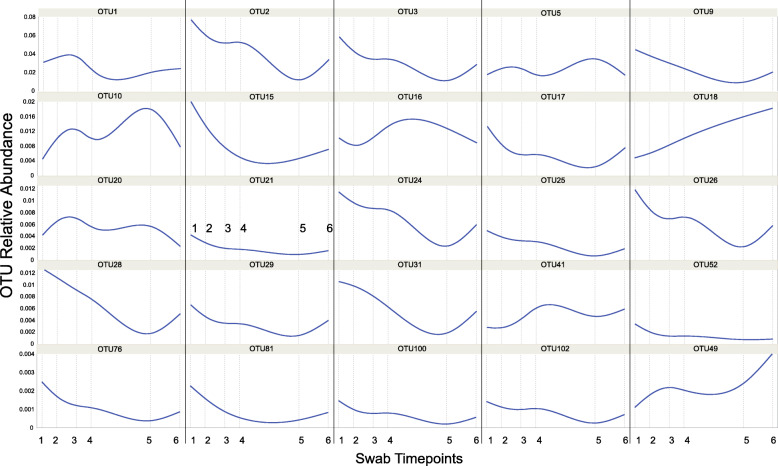
Core OTUs relative abundances over time using Kernel Smoothing (Loess) estimations. Dashed vertical lines represent sampling time points (days 0, 3, 7, 10, 22, 28 – see Fig. 1). Notice changing y-axes values for relative abundances

The core community (OTUs found in at least 90% of all samples) consisted of 25 OTUs, 4 which were present on 100% of the frogs at each sampling time (Table 4). All of the core taxa shifted in relative abundance over time (Table 4; Fig. 4) often with oscillating abundances (Fig. 5). The average relative abundance of the core community over sampling timepoints 1–6 were 2.15, 3.05, 1.46, 2.02, 1.02, and 1.10%, respectively. The representative sequences of these core OTUs were compared against a database of amphibian skin-associated bacteria with known interactions (antagonistic or faciliatory) with *Bd* [10] using BLASTn (https://www.ncbi.nlm.nih.gov). Using a 99% sequence similarity threshold to consider OTUs as *Bd-*associated, seven of the 25 core OTUs are *Bd* inhibitors (28%) and one is a known *Bd* enhancer (4%) (Table 4; Table S1). Interestingly, *Btk* 72 was not a core taxon and did not change in abundance with treatments (F_3,152_ = 0.567, *P* = 0.637). *Btk 72* was present on the skin of individuals in every treatment group prior to exposures. However, our Kendall Tau associations (Table S1) suggests that the presence of *Btk* 72 is significantly positively associated with 32% of the core OTUs (8 of 25) and was not negatively associated with any core OTUs, suggesting that several core taxa may facilitate *Btk 72* or have other enhancing capabilities.

**Table 4:** The taxonomic classification of core OTUs (> 90% of all samples) based on RDP classification (v.10). The results of repeated measures ANOVA of the relative abundance of each OTU is also included. F statistics are included with degrees of freedom (F_df, dfDen_) where dfDen is the denominator df based on Kenward-Roger first order approximations with Kacker-Harville correction. * *p* < 0.05, ** *p* < 0.01, *** *p* < 0.001. Where significant, partial *η*^2^ effect sizes are presented parenthetically

Phylum (Order)	Family (Genus)	OTU #	Treatment	Time	Treatment x Time
Actinobacteria					
Actinomycetales	Corynebacteriaceae (*Corynebacterium*)	76	F_3,23.2_ = 2.364	***F***_***5,101.7***_ = 13.511***(0.354)	F_15,100.9_ = 1.305
	Dermacoccaceae (*Dermacoccus*)	28	F_3,22.3_ = 1.183	***F***_***5,100.0***_ = 29.749***(0.547)	***F***_***15,99.3***_ = 2.643**(0.244)
	Micrococcaceae (*Micrococcus)*	31^i^	F_3,23.4_ = 1.482	***F***_***5,106.2***_ = 30.343***(0.552)	F_15,105.8_ = 1.459
	Micrococcaceae (*Nesterenkonia*)	2^a^ 24	F_3,18.9_ = 0.454 F_3,18.9_ = 0.409	***F***_***5,103.6***_ = 23.845***(0.492) ***F***_***5,100.0***_ = 23.353***(0.487)	***F***_***15,103.2***_ = 3.206***(0.281) ***F***_***15,99.6***_ = 3.253***(0.284)
	Propionibacteriaceae (*Propionibacterium*)	9	F_3,18.9_ = 1.270	***F***_***5,99.2***_ = 16.822***(0.406)	***F***_***15,98.1***_ = 3.849***(0.319)
Bacteriodetes					
Flavobacteriales	Flavobacteriaceae (*Flavobacterium*)	15 81	F_3,22.1_ = 0.478 F_3,17.6_ = 0.396	***F***_***5,99.5***_ = 9.886***(0.288) ***F***_***5,89.2***_ = 12.982***(0.345)	F_15,99.0_ = 1.387 F_15,88.6_ = 0.877
Firmicutes					
Bacillales	Bacillales incertae sedis (*Caldalkalibacillus*)	3^a^ 26^a^ 102	F_3,22.1_ = 1.657 F_3,22.3_ = 2.954 F_3,19.7_ = 0.182	***F***_***5,107.6***_ = 20.691***(0.456) ***F***_***5,109.3***_ = 19.829***(0.446) ***F***_***5,89.9***_ = 9.516***(0.279)	***F***_***15,107.2***_ = 2.807***(0.255) ***F***_***15,108.8***_ = 2.068*(0.201) ***F***_***15,88.7***_ = 2.069*(0.201)
	Bacillaceae (*Bacillus*)	29	F_3,21.4_ = 1.246	***F***_***5,10,442***_ = 16.592***(0.402)	***F***_***15,103.9***_ = 1.998*(0.196)
	Bacillaceae (*Geobacillus*)	100	F_3,20.2_ = 2.921	***F***_***5,93.3***_ = 13.386***(0.352)	F_15,92.6_ = 1.267
Proteobacteria					
Burkholderiales	Alcaligenaceae (*Bordetella*)	1^i^	F_3,22.2_ = 0.304	***F***_***5,101.9***_ = 17.454***(0.415)	F_15,101.6_ = 0.889
	Comamonadaceae (*Delftia*)	16^i^	F_3,25.0_ = 0.793	***F***_***5,107.8***_ = 14.084***(0.364)	***F***_***15,107.4***_ = 2.077*(0.202)
	Comamonadaceae (*Comamonas*)	18^i^	F_3,23.2_ = 1.510	***F***_***5,103.1***_ = 7.340***(0.229)	F_15,102.3_ = 1.731
	Comamonadaceae (*unclassified*)	41	F_3,22.6_ = 1.869	***F***_***5,103.7***_ = 9.564***(0.281)	F_15,103.5_ = 1.111
Pseudomonadales	Moraxellaceae (*Acinetobacter*)	5^i^ 20^e^	F_3,22.1_ = 0.498 F_3,24.7_ = 0.724	***F***_***5,105.7***_ = 11.316***(0.315) ***F***_***5,100.7***_ = 11.135***(0.311)	F_15,105.3_ = 1.139 F_15,100.5_ = 1.202
	Moraxellaceae (*Enhydrobacter*)	25	***F***_***3,22.6***_ = 5.52**^§^**(0.118)**	***F***_***5,109.2***_ = 12.433***(0.335)	F_15,107.7_ = 1.397
	Pseudomonadaceae (*Pseudomonas*)	10^i^	F_3,21.6_ = 0.634	***F***_***5,97.7***_ = 7.911***(0.243)	F_15,96.8_ = 0.931
Oceanospirillales	Halomonadaceae (*Halomonas*)	17^a^	F_3,23.3_ = 1.485	***F***_***5,108.9***_ = 26.467***(0.518)	***F***_***15,108.5***_ = 2.392**(0.225)
Enterobacteriales	Enterobacteriaceae (*Cronobacter*)	21^i^	F_3,21.9_ = 0.623	***F***_***5,102.2***_ = 12.679***(0.340)	F_15,101.8_ = 1.091
Methylophilales	Methylophilaceae (*Methylophilus*)	49	F_3,20.7_ = 2.291	***F***_***5,94.0***_ = 5.107***(0.172)	F_15,93.4_ = 1.047
Sphingomonadales	Sphingomonadaceae (*Sphingomonas*)	52	F_3,22.3_ = 1.350	***F***_***5,99.8***_ = 18.892***(0.434)	F_15,99.3_ = 1.227

a = OTUs present in all frogs at all time points

i = OTUs with ^3^99% sequence similarity for *Bd-*inhibiting bacteria (Woodhams et al. 2015)

e = OTUs with ^3^99% sequence similarity for *Bd*-enhancing bacteria (Woodhams et al. 2015)

§=Treatment effect - Tukey HSD indicates Bd (A), BB (AB), N (B), Btk (B) where A > B

Additionally, to identify biomarker taxa that are more abundant in certain treatment groups for each sampling date, we used a Linear Discriminate Analysis (LDA) effect size (LEfSe). In doing so (Table S2), we identify several OTUs that are biomarker throughout the experiment, many of these are rare OTUs and unlikely to play major roles in overall community function. We identify 28 biomarker OTUs for Timepoint 1 (T1; 17 for N, 6 for BB, 3 for Btk, and 2 for Bd), 18 for T2 (3 for N, 6 for BB, 3 for Btk, an 6 for Bd), 18 for T3 (0 for N, 12 for BB, 2 for Btk, and 4 for Bd), 28 for T4 (1 for N, 4 for BB, 11 for Btk, and 12 for Bd), 35 for T5 (5 for N, 8 for BB, 7 for Btk, and 15 for Bd), and 23 for T6 (11 for N, 4 for BB, 5 for Btk, and 3 for Bd). This suggests that time x treatment interactions play large roles in differential OTU abundances across our experimental framework.

## Discussion

This study is the first to examine how cutaneous microbial communities on *Lithobates sphenocephalus* respond to biotic perturbations over time and demonstrates that these communities, while impacted in the short-term, are highly resilient to biotic disturbances, including short-term exposures to concentrated bacterial cells and *Bd* zoospores. We confirmed that *Bd* infection was successful as the treatment group exposed to *Bd* exhibited 100% infection prevalence. The lack of shifts in diversity with treatments was surprising as *Bd* infection has been associated with a disruption of the amphibian microbiome in *Rana sierrae* [17, 23]. Jani & Briggs found that the most disruption occurred when infection loads were above 1000 *Bd* cells/swab [17]**.** While our *Bd* infection levels were measured in genomic equivalents per ml, the amphibians exposed to *Bd* exhibited high average infection loads (above 1000 genomic equivalents/ml) throughout the study with minimal microbiota disruption. Additional work is needed to confirm whether *Bd* is disruptive to the microbiome of *L. sphenocephalus* as it is possible that rearing and housing conditions significantly affect this response [33], the *Bd-*associated disruption of the microbiome is host-species dependent, or disruption is contingent on the *Bd* strain causing infection. Surveys of wild *R. sierrae* populations and lab experiments suggested that *Bd* causes extreme disturbance to the microbiome which may be associated with epizootic die-offs [23], whereas *L. sphenocephalus* can maintain *Bd* infections in the population without declines [32]. In our study, it does not appear that *Bd* was heavily disruptive to the microbiome for *L. sphenocephalus,* as communities on infected individuals differed minimally from those on uninfected individuals. The resilience demonstrated by the *L. sphenocephalus* microbiota in this study may play a role in their resistance to chytridiomycosis even when carrying *Bd* skin infections. It is important to note that we sampled frogs in controlled environments with very little opportunities for additional microbial inputs onto frog cutaneous membranes; it remains to be seen how these biotic disturbances impact wild populations. While we acknowledge that amphibian bacterial communities in captivity are vastly different from those found in natural systems, this recovery towards a pre-disturbance state without inputs from environmental reservoirs suggests that the core community that colonizes early in life may remain with the animal, regardless of the environmental reservoir present during disturbance. Loudon et al. [11] found that salamanders housed in sterile soil in captivity still retained specific bacterial groups. Even in the wild, four geographically isolated populations of *Ensatina eschscholtzii xanthoptica* maintained a similar core microbiome that differed from their environmental reservoir [34]. Additionally, microbial communities on the skin of amphibians can be highly amphibian species-specific [16]. Different amphibian species housed together exhibit unique microflora with very little overlap [16, 35]. Thus, it is possible that other host-specific factors may be involved in maintaining the microbial community during *Bd* infection, contributing to robust microbial communities for some amphibian species and more susceptible communities in others. For instance, antimicrobial peptides (AMPs) are produced and excreted onto the surface of the skin. AMPs are genetically determined, some are effective against *Bd,* and thought play a role in the microbial species that are permitted to live on the skin [36].

Our hypothesis that *Btk* exposure would increase the abundance of *Btk* on the skin was not supported. Instead, we found that many individuals were already carriers of this bacterial isolate (*Btk* 72). However, *Btk* 72 was associated with higher abundances of some genera of bacteria that include known *Bd-*inhibitors (Table S1). While *Btk* 72 appears to already be a common constituent of the *L. sphenocephalus* microbiome, it may not be in high enough abundance to facilitate anti-*Bd* activity as observed in in vitro studies (Weeks & Parris, unpublished). Nevertheless, the presence of *Btk* in the community may promote the growth of other species known to inhibit *Bd.* Individuals in the *Bd* treatment group experienced 100% infection prevalence while those that were exposed to *Btk* prior to *Bd* only exhibited 60% infection prevalence, which suggests that *Btk* may directly or indirectly alleviate *Bd* establishment. However, more research needs to be done to confirm this potential. We also found that seven of the core OTUs in our study shared at least a 99% sequence similarity to other skin-associated amphibian OTUs with demonstrated anti-*Bd* activity ([10]; Table 4, Table S1)*.* These OTUs included members of the genera *Micrococcus, Bordetella, Delftia, Comamonas,* and *Acinetobacter.* Interestingly, the sequences found among our core OTUs did not match any of the sequences obtained from Holden et al. [21], even though they characterized the culturable microbiome from *L. sphenocephalus* juveniles from the same population. A potential reason for sequence-specific differences could be the age of the amphibians; Holden et al. [21] sampled juveniles while we sampled from adults. Ontogenetic changes occur in antimicrobial peptides (AMPs) that are secreted onto the skin in *L. sphenocephalus*, which may alter the cutaneous microbiome differently for frogs of different ages*.* For instance, AMPs that are secreted onto the skin of amphibians change throughout development and juveniles do not express a mature profile of AMPs until ~ 12 weeks post-metamorphosis [37].

The frogs in all experimental groups had abundant core OTU membership, with the relative abundance shifting for many during the experiment, but treatment was rarely a significant factor in the observed shifts (Table 3; Table 4; Fig. 5). Additionally, many core OTUs trended toward pre-experimental abundances by the end of the experiment (Fig. 5). The 4 OTUs found on every frog included members of *Halomonas, Caldalkalibacillus,* and *Nesterenkonia.* All of these OTUs, which are commonly isolated from water and soil samples in the environment, initially decreased in abundance but returned to a similar relative abundance at the end of the experiment. The presence of some core OTUs in the community may play a foundational role, producing antifungal compounds or facilitating the growth of other microorganisms that do. For some amphibian species that are resistant to *Bd* infections or chytridiomycosis, the presence of keystone species that facilitate a more resilient, protective community may contribute to disease resistance [38]. *Janthinobacterium lividum* produces anti-*Bd* metabolites, has been used as probiotic against *Bd* [7, 39], and is commonly found in the microbiome of some amphibian taxa [40, 41]. For many susceptible amphibians, it is a successful anti-*Bd* probiotic. Unfortunately, *J. lividum* is unlikely to be effective for every amphibian species [42], so it is important to identify other candidate keystone species that may be vital to the microbiome of a healthy amphibian population, even if in the absence of direct anti-*Bd* activity in vitro. For *Lithobates sphenocephalus,* core OTUs may act as similar keystone species which may aid communities in recovery from disturbances. Probiotic therapy with keystone species may be a long-term solution to restore a dysbiotic community to a protective, pre-disturbance composition [43].

Microbial diversity estimators exhibited major shifts across sampling time throughout the experiment. Microbial richness and diversity on frogs were not sensitive to treatment but were highly dynamic over time (Table 1; Fig. 2). Evenness, however, was sensitive to treatment as frogs in groups exposed to *Bd* (Bd and BB) exhibited more similar evenness with each other than to individuals in the control or *Btk* group (Table 1; Fig. 2). The significant shift in Simpson’s diversity at sampling time 2 was seen in all treatment groups (Fig. 2) and is likely a disturbance from handling the frogs during this experimental step. All treatment groups experienced a significant drop in diversity following this step but returned to pre-treatment levels within a few days. Subsequent handling and exposure baths throughout the remainder of the experiment did not appear to affect diversity estimates or community composition. Recovery from handling disturbance is particularly important to understand for captive breeding programs and researchers that handle sensitive species. In one study, equipping frogs with passive integrated transponders caused disruption of bacterial communities, but recovery to a pre-disturbance composition took place after 2 weeks [14]. If the microbial community for a threatened amphibian species is more prone to an inability to recover from a handling disturbance, investigators and breeders may alter handling protocols and consider follow-up with probiotic or prebiotic therapy (e.g. diet enrichment). For instance, carotenoid-enriched diets in captivity supported a greater species richness and abundance of bacteria on *Agalychnis callidryas* (Red-Eyed Tree Frogs) [44]. Furthermore, species reintroduction from captive breeding programs would benefit from characterizing recovery potential of microbial communities.

Many other factors can affect the cutaneous microbiome of amphibians including captivity [45], diet [44], seasonality [46], and internal parasites [13]. Stability of a microbial community, and ability to recover from perturbations, can be determined by changes in the community composition following small environmental changes [47]. Over time, *L. sphenocephalus* communities recovered after initial perturbations to be more similar, though not indistinguishable, to pre-treatment communities as evidenced by NMDS, PERMANOVA, and AWOrD analyses (Fig. 3, Table 2 and 3). This demonstrates that bacterial residents were robust and began recovery trajectories quickly following biotic disturbances (*similar to* [48]). Other potential microbial community responses could have included an alternative stable state that does not resemble the pre-treatment state [47] or a dysbiotic state that may result in more dispersed, variable microbiomes. While an alternative state is stable, it may alter any protective function (reviewed in [12]). For instance, the *Bd-*associated community disruption observed in *Rana sierrae* [17, 23] could be the result of poor community recovery from *Bd* infection. Subsequent dysbiosis or an alternative stable state may no longer provide protective properties. Additionally, the stability of keystone core OTUs over time may be a significant contributor to stability of the community [37].

## Conclusion

This study revealed that the amphibian species *Lithobates sphenocephalus* harbors a resilient cutaneous microbiome in which communities recovered to resemble the pre-disturbance state following two biotic disturbances. The recovery to a pre-disturbance state may be a key component in *Bd* disease resistance for some amphibian species. For amphibian species that are known to experience population declines from *Bd,* cataloging microbiome response to disturbances (both *Bd* and otherwise) would complement use of anti-*Bd* probiotics. The addition of a probiotic may not be protective if a population is already experiencing microbial community instability. The resilience, or lack thereof, in the host microbial community as a whole may allow researchers to better predict which populations are susceptible to *Bd* die-offs.

## Materials and methods

### Animal Collection & Husbandry

Following breeding events in March 2016, partial egg masses (*n* = 7) of *L. sphenocephalus* were collected from three populations at Edward J. Meeman Biological Field Station (MBFS, The University of Memphis) in Shelby County, Tennessee, USA (35°23′22.66″ N, 90°02′15.75″ W). Eggs were transported to the MBFS laboratory and kept in aquaria with mesocosm water (described below) with oxygen bubblers until hatching. Upon hatching and reaching free-swimming stage (Gosner 25 [49];), individuals were fed a 3:1 mixture of crushed Kaytee rabbit chow and Tetramin® tropical flakes ad libitum with a 14:10 h light:dark cycle. After 2 weeks, tadpoles were transferred to randomized outdoor mesocosm tanks (*n* = 15 per tank) at MBFS. The mesocosms consisted of 1000 L polyethylene tanks filled with water from the MBFS facility to a depth of 30.5 cm (~ 613 L), 300 g of dried leaf litter (primarily *Quercus* species), and a 100 ml dose of concentrated zooplankton suspension collected from a local pond [50]. Mesocosm algal and zooplankton communities were given two full weeks to develop before the addition of tadpoles.

At Gosner 42 (emergence of front limbs), individuals were removed from mesocosms and maintained in individual 1.5 L plastic containers with autoclaved sphagnum moss to maintain moisture. Containers were cleaned twice a week (replaced soiled water) and frogs were fed calcium-dusted crickets. Until experimental exposures began, frogs were maintained at 19 °C on a 12:12 light:dark hour photoperiod.

### *Btk* culture preparation

To prepare the bacterial bath used in this study, *Bacillus thuringiensis subsp. kurstaki* was sub-cultured from Monterey B.t. liquid biocide (Lawn and Garden Products, Fresno, CA) with an inoculating loop and streaked onto 1% tryptone agar plates. After incubating plates at room temperature for 48 h, visible colonies were transferred to 5 ml tubes of autoclaved 1% tryptone broth. Broth cultures were incubated at 30 °C on an orbital shaker for 28 h to reach stationary growth phase (OD_600nm_ = 0.84 ± 0.01, OD targets based on preliminary research). Following incubation, 1 ml of the liquid cultures were centrifuged at 4500×g for 10 min, the supernatant was discarded, and the cells were resuspended in sterile water. This process was repeated three times. To enumerate colony forming units (CFU), a 0.1 ml aliquot of 10^− 8^ diluted stock culture was plated onto 1% tryptone agar with an agar overlay. Following 48 h incubation at 30 °C, colonies were quantified from 3 plates and averaged. The final concentration of the stock solution used in exposures was 3.4 × 10^11^ CFU/ml.

### *Bd* preparation

*Batrachochytrium dendrobatidis* culture was prepared using a strain (FMB 003 [51];) isolated from a local, infected *L. sphenocephalus* adult in 2010 at Meeman-Shelby State Park, Shelby County, Tennessee, USA. Plates were prepared on 1% tryptone agar from 2 ml of homogenized *Bd* stock culture, sealed, and incubated at room temperature for 10 d. Following incubation, plates were flooded with 3 ml of aged tap water for 45 min to harvest zoospores. The zoospores were pooled from the plates and counted using a hemocytometer and the final concentration used was 2 × 10^6^ zoospores/ml.

### *Btk* and *Bd* exposures

*L. sphenocephalus* adults (33 weeks post-metamorphosis) were randomly assigned into four treatment groups: N (negative control; *n* = 6), *Btk* (*B. thuringiensis kurstaki* exposure; *n* = 8)*, Bd* (*B. dendrobatidis* exposure; *n* = 7)*,* BB (*Btk* + *Bd; n* = 10). Individuals had been housed in the 1.5 L plastic containers with sphagnum moss for ~ 30 weeks prior to treatments. The day prior to experimental treatments, they were rinsed with 30 ml of autoclaved aged tap water to remove transient bacteria and placed into new bleach-sterilized plastic containers (1.5 L) with autoclaved sphagnum moss and 20 ml of autoclaved aged tap water. During the experiment, individuals were removed for weekly cage cleanings, but remained segregated in new zip top plastic bags (1 qt). Crickets were added to the container following cage cleaning.

All individuals in both control and experimental groups experienced the same amount of handling during the experiment. Individuals in treatment groups *Btk* and BB were exposed to two inoculation baths of *Btk.* Individuals in the BB group were later exposed to *Bd.* The first *Btk* exposure, on Day 0 (see Fig. 1), was diluted from the stock solution (3.4 × 10^11^ CFU/ml) to 1.7 × 10^11^ CFU/ml by adding 5 ml of stock solution and 5 ml of sterile water to sterile 120 ml sample cups with air holes in the lid. The second exposure (Day 7) was diluted from the same stock solution. Due to limited stock volume, 4 ml of *Btk* stock was combined with 11 ml of sterile water for a final exposure concentration of 9.1 × 10^10^ CFU/ml. Individuals in the *Bd* and N groups were exposed to the same volume of sterile water. After 24 h in the bath, individuals were placed back into their containers. We used two inoculation baths to mimic the multiple exposures that amphibians experience in nature. Following *Btk* exposures, frogs were not disturbed for 1 week, aside from cage cleaning, to allow the potential for *Btk* colonization.

For *Bd* exposures, individuals in the *Bd* and BB groups were exposed to 6 × 10^5^ zoospores/ml of *Bd* in 30 ml for 24 h (Day 14; Fig. 1). Frogs in the N and *Btk* group were exposed to water collected and diluted from 1% tryptone agar plates as a negative control for the residual tryptone media. Mass was measured while individuals were inside of zip top plastic bags with a Pesola Spring scale (20 g ± 0.2) and snout-vent length (SVL) was measured with SPI Dial Calipers (± 0.1 mm). The measurements taken at timepoint 1 and timepoint 6 are presented in Table 5. There were no significant differences in mass or SVL for any of the experimental groups between timepoint 1 and timepoint 6.

**Table 5:** Average mass and SVL for each experimental group at timepoint 1 and timepoint 6

	Timepoint 1		Timepoint 6	
	Mass (g) (mean ± s.e.)	SVL (mm) (mean ± s.e.)	Mass (g) (mean ± s.e.)	SVL (mean ± s.e.)
N	4.85 ± 0.49	37.3 ± 1.08	4.78 ± 0.48	38.5 ± 1.03
Btk	4.48 ± 0.12	37.4 ± 0.57	4.76 ± 0.18	38.6 ± 0.56
Bd	5.34 ± 0.22	39.4 ± 0.57	5.36 ± 0.15	40.3 ± 0.71
BB	4.36 ± 0.23	37.1 ± 0.58	4.55 ± 0.21	38.1 ± 0.73

### Sampling and DNA extractions

To assess cutaneous bacterial communities and *Bd* infection status, samples were taken via sterile cutaneous swabbing. During swab collection (see Fig. 1), each frog was handled with a fresh glove and swabbed five times each on the ventral posterior patch, dorsal surface, and ventral side of both hind legs with a sterile cotton swab. A total of 6 swabs were taken for each frog (*n* = 186) over the experiment at various timepoints. These include: Day 0 (Timepoint 1), Day 3 (Timepoint 2), Day 7 (Timepoint 3), Day 11 (Timepoint 4), Day 23 (Timepoint 5), and Day 29 (Timepoint 6). Swabs were then stored at − 20 °C in 1.5 ml sterile microcentrifuge tubes until DNA was extracted using the DNeasy Blood and Tissue DNA Extraction Kit (Qiagen, Valencia, CA USA, animal tissue protocol). DNA was stored at − 20 °C until molecular work was conducted.

### NGS library preparation and sequencing

Sequencing libraries were generated by selectively amplifying the bacterial 16S (V4) region using a two-step amplification process (*following* [52]). Briefly, the V4 region of the 16S rRNA gene repeat was amplified using the primer pairs nexF-N [3–6]-515f and nexR-N [3–6]-806r where 515f and 806r [53] are bacteria gene primers, N [3–6] represents four identical primers with the exception of containing a range of ambiguous nucleotides (3–6) mixed to equal molarity to increase nucleotide diversity during sequencing, and nexF and nexR are Nextera forward and reverse sequencing primers. PCRs were conducted in 20 μL reactions using 2 μL extracted DNA, 4 μL 5X Phusion High-fidelity Buffer, 200 μM each dNTP, 1 μM of each forward and reverse primer, 0.2 μL Phusion HotStart II DNA Polymerase (ThermoFisher Scientific; Waltham, MA, USA), and 7.8 μL molecular grade H_2_O. Primary PCR parameters were 98 °C for 30 s, 25 cycles of 98 °C for 20 s, annealing temperature for 30 s at 52.5 °C, and 72 °C for 40 s, followed by a final extension at 72 °C for 10 min, all ramp rates were 1 °C/second (SimpliAmp Thermal Cycler, Applied Biosystems, Foster City, CA, USA). This resulted in a final 1° PCR construct of nexF-N [3–6]-primer-{V4}-primer-N [3–6]-nexR. After primary PCR generation, secondary PCR reactions were conducted in 25 μL reactions using forward primers that include the P5-i5-overlap and the reverse primers P7-i7-overlap where P5 and P7 are the Illumina Adaptor sequences, i5 and i7 are 8 bp unique Molecular Identifiers (MIDs – barcode), and the overlap is the partial nexF and nexR sequences that acts as the annealing site for the 2° PCR and prevents additional amplification of bacterial DNA that does not have the artificial overlap sequences. The forward and reverse barcoded 2° primers were mixed in a combinatorial fashion to generate unique dual barcoded primers (see Table S3 for primer and MID information) in a working concentration of 10 μM (5 μM for each primer). The 2° PCR reactions contained 2.5 μL of 1° PCR product, 5 μL 5X Phusion High-fidelity Buffer, 200 μM each dNTP, 0.5 μM of each forward and reverse primer, 0.25 μL Phusion HotStart II DNA Polymerase (0.02 U/μL final concentration; ThermoFisher Scientific), and 7.5 μL molecular grade H_2_O with the PCR parameters of 95 °C for 2 min, 8 cycles of 95 °C for 20 s, 50 °C for 20 s and 72 °C for 50 s, followed by a final extension at 72 °C for 10 min. This produced the final amplicon constructs of P5-i5-nexF-N [3–6]-primer-{V4}-primer-N [3–6]-nexR-i7-P7 using a total of 32 cycles.

Secondary PCR products were cleaned using Axygen AxyPrep Mag PCR clean up beads (Axygen Biosciences, Union City, CA, USA) following kit protocol with the modification using a 1:1 bead solution to reaction volume ratio to better select against small fragments and primer-dimers [48]. Cleaned PCR products were quantified using Qubit 3.0 fluorometric assays (dsDNA HS Assay Kit; ThermoFisher Scientific). PCR products were pooled into a library at equal concentrations and sequenced on one Illumina MiSeq (v.3, 300PE) at the Kansas State University Integrated Genomics Facility (Manhattan, KS, USA). Demultiplexing of the raw sequence data using the unique i5 and i7 sequence combinations provided individual paired fastq files. Sequence data is deposited in SRA at NCBI (**BioProject PRJNA646730, BioSamples SAMN15560190-SAMN15560352**).

### *Btk* reference sequencing and *Bd* DNA infection confirmation

To determine which operational taxonomic unit (OTU; see below) corresponds to the strain of *Btk* used in exposures, a representative sequence from *Btk* was obtained. DNA from *Btk* cultures were extracted using the DNeasy Blood and Tissue DNA Extraction Kit and PCR was conducted in 50 μL reactions targeting the partial 16S region (357F and 1100R [54];), which fully encompasses the V4 region used in our community analyses. PCR parameters consisted of 98 °C for 30 s, 25 cycles of 98 °C for 20 s, annealing temperature for 30 s at 58 °C, and 72 °C for 40 s, followed by a final extension at 72 °C for 10 min. PCR product was visualized via gel electrophoresis (1% agarose in TBE) and cleaned using DNA Clean & Concentrator kits following protocols (Zymo Research, Irvine, CA, USA and Sanger sequenced (10 ng DNA, and 0.5 μL or each primer [10 μM]) at the University of Tennessee Health Science Center (Memphis, TN, USA) and the representative sequence is deposited in GenBank (accession **MT771634**).

To assess *Bd* infection status, real time quantitative PCR (qPCR; Bio-Rad CFX96 Touch Real-Time PCR Detection System) was used to quantify *Bd* DNA loads from each sample (following [55]). PCRs were conducted in 20 μL reactions with 900 nM of the PCR primers ITS1–3 Chytr and 5.8S Chytr [55], 240 nM MGB probe, TaqMan Exogenous Internal Positive Control, and 250 nM DyNamo Flash Probe. The qPCR protocol for DyNamo Flash Probe was used to set PCR parameters (Initial denaturation: 95 °C for 7 min, Annealing/Extension: 60 °C for 30 s) and included *Bd* DNA and sterile water for positive and negative controls, respectively. A plasmid standard dilution series (Pisces Molecular, Boulder, CO, USA) was used to quantify zoospore genomic equivalents and all reactions were run in duplicate.

### *Btk* interactions with other taxa

To identify which obtained OTU matched the *Btk* strain used in our inoculations, we examined representative sequences of each demarcated OTU and compared them (BLASTn) to the Sanger sequence obtained of the isolated *Btk* used for exposure experiments. OTU72 (hereafter referred to as *Btk* 72) was locally identical (100%) to the *Btk* strain. To investigate if *Btk* 72 co-associates with (positive correlation) or is mutually exclusive of (negative correlation) core obtained OTUs (see below), Kendall Tau correlations were determined between *Btk 72* and all core OTU sequence abundances and alpha levels for significance were adjusted based on Šidàk corrections (adj. α = 0.002) for multiple comparison corrections. All statistics were conducted using a combination of R, JMP Pro (v.14), and mothur. We also used regression analysis to assess any associations between the *Btk* abundance (OTU72 - see below) and *Bd* genomic equivalents.

### Bioinformatics

Illumina Sequences were processed using the program mothur (v.1.40 [56];). The forward and reverse sequences were contiged and screened to cull any sequences with ambiguous bases, or greater than 10 homopolymers and merged into a single FASTA file and trimmed to eliminate primer sequences. Sequences were aligned against the SILVA (release 132) reference alignment and filtered to exclude non 16S V4 regions. Sequences were preclustered to remove basepair variation due to sequence chemistry errors (using pseudo-single-linkage clustering following [57] as implemented in mothur), screened for chimeras (mothur implemented VSEARCH [58];), and putative chimeras were culled. Sequences were screened for off-target amplification (non-bacterial in origin) by classifying all sequences using a mothur implemented Naïve Bayesian Classifier [59] against the RDP training set (v.10). Non-target lineages were culled and distance matrices (fully aligned distances not punishing terminal gaps) were generated. Sequences were clustered into operational taxonomic units (OTUs) at a 3% dissimilarity threshold using OptiClust [60]. OTUs with fewer than 10 sequences globally were eliminated to prevent inclusion of potentially spurious OTUs into analyses [61, 62]. After all sequence quality control 2343 OTUs were retained. Negative controls (ddH2O) were included throughout extraction and amplicon generation and remained free of visible contamination during amplification, were eliminated completely during these sequence cleanup steps. OTU distributions within each sample, full RDP taxonomy strings along with bootstrap support, and representative sequences of each delineated OTU are presented in Table S4.

### Diversity indices

Diversity estimates based on an iterative subsampling approach using 1000 iterations at a subsampling depth of 5635 sequences per sample (without replacement - selected to retain all samples) were generated (in mothur) and the average estimates were used for all downstream analyses. These include observed OTU richness (*S*_*obs*_), the complement of Simpson’s diversity (*1-D*; 1 - ∑*p*_*i*_^2^) where *p*_*i*_ is the frequency that each OTU occurred in each sample, Simpson’s evenness (*E*_D_: (1/*D*)/*S*_*obs*_) where *D* is Simpson’s. Diversity estimators were analyzed to examine if treatments impact diversity using a repeated measures analysis of variance (ANOVA) with Kenward-Roger first order approximations with Kacker-Harville corrections which allows for partial degrees of freedom. To estimate the effect size of any significant relationships, we calculated partial eta-squared (partial *η*^2^) for repeated measures ANOVA (*following* [63]).

### Community dynamics

To test if bacterial communities differ across treatments, time, and their interactions, a permutational multivariate analysis of variance (PERMANOVA [64];) of average Bray-Curtis dissimilarity values (iteratively subsampled as above) was conducted in the program R (v.3.3.3) with the package *vegan* (function *adonis* with 999 iterations*, strata* = individual frog to facilitate repeated measures, [65]) and post-hoc multiple comparisons were examined using the package *RVAIDeMemoire* (function *pairwise.perm.manova* with FDR corrections, 999 iterations, [66]). To visualize communities, nonmetric multidimensional scaling (NMDS) was conducted (using mothur) using 1000 iterations and optimally resolved to across 4 axes (4D stress = 0.1544). Further, to examine community shifts on individual frogs after perturbation events to test community recovery over time, we used the AWOrD metric (Axis Weighted Ordination Distance [67];). The AWOrD quantifies distance in ordination space (NMDS here) between any two samples across multiple axes whilst accounting for ordination axes coefficients of determination (scaled by R^2^ for each axis) and is based on a modified Manhattan distance. If AWOrD values are lower, then two samples are highly similar whereas if values are higher, they are more dissimilar. We calculated AWOrD values for the four solved NMDS axes between sampling timepoint one (T1) and all subsequent sampling timepoints for each individual frog and using two-way ANOVAs, we tested if AWOrD values differed between treatments, across time (T1 vs T2, T1 vs T3, …, T1 vs T6), and treatment by time interactions.

Further, we sought to examine patterns of common core taxa (OTUs) across our experimental framework. To identify core taxa, we compiled a list of OTUs that are present in at least 90% of the frogs in our experiment. Core OTUs were tested using repeated measures ANOVA (relative abundance, logit transformed, sampling effort [time] as a categorical variable) to examine if these core taxa change in abundance across treatments, time, or their interactions with Kenward-Roger first order approximations with Kacker-Harville corrections. When treatment had a significant effect, Tukey HSD post-hoc tests were conducted to identify how treatments differ and effect sizes were determined using partial eta-squared (partial *η*^2^). Additionally, to visualize changes in the relative abundances of core taxa over time (continuous), we fit Kernel Smoothing (Loess [68];) lines using linear local fits, tri-cube weighing, and four iterations to derive best fit lines. Further, to determine if our obtained core taxa putatively inhibit, facilitate, or have no effect on *Bd*, we compared our core OTUs to sequences obtained from the functional analysis by Woodhams et al. [10] using BLASTn and identified our OTUs as a match if Query Coverage =100% AND Identity ≥99%.

Since we observed significant community and OTU based time by treatment interactions (see results), we aimed to identify biomarker OTUs for treatment conditions for each timepoint. To do so, we used the mothur implementation of LEfSe [69] and identified biomarker OTUs separately for each sampling timepoint after Kruskal Wallis and Wilcoxon tests to determine a signed LDA log-score and associated *p*-values.

## Supplementary information

**Additional file 1: Table S1.** Results of co-association analysis between *Btk* 72 and all core OTUs presented along with genus identifications, correlation coefficients, and *p*-values. Also presented are BLASTn matches of core OTUs to *Bd* associated taxa from Woodhams et al. 2015, with % identity and max bit scores.

**Additional file 2: Table S2**. Results of LefSe biomarker identification for each timepoint (T1-T6) as reflected in Fig. 1. Presented are significant biomarker OTUs, with LogMaxMean values, Class (treatment OTUs are biomarkers of), LDA and associated p-values.

**Additional file 3: Table S3**. Primer sequences and MID (i5 and i7) sequences used for parsing sequences into experimental units.

**Additional file 4: Table S4**. OTU taxonomic identification, representative sequence and full OTU by sample sequence count data are presented along with total number of sequences of each OTU.

## Data Availability

All data generated during this study are available in the manuscript or supplementary files. Sequence data have been submitted to Sequence read archive at NCBI under **BioProject PRJNA646730, BioSamples SAMN15560190-SAMN15560352**) **and GenBank (MT771634)**.

## Abbreviations

AMP: Antimicrobial Peptide
N: Negative control group
AWOrD: Axes Weighted Ordination Distance
NMDS: Non-metric Multidimensional Scaling
BB: Group exposed to *Bd* and *Btk*
OTU: Operational Taxonomic Unit
*Bd*: *Batrachochytrium dendrobatidis*
PERMANOVA: Permutational multivariate analysis of variance
*Btk*: *Bacillus thuringiensis kurstaki*
RDP: Ribosomal Database Project
LEfSe: Linear Discriminate Analysis Effect Size
MBFS: Edward J. Meeman Biological Field Station
