# Supplementary material for: Recovery and resiliency of skin microbial communities on the southern leopard frog (Lithobates sphenocephalus) following two biotic disturbances
Source: Anim Microbiome. 2020 Sep 22;2:35. doi: 10.1186/s42523-020-00053-5 (PMC7807490; doi:10.1186/s42523-020-00053-5)
Supplement: Supplementary file 1 — Additional file 1: Table S1. Results of co-association analysis between Btk 72 and all core OTUs presented along with genus identifications, correlation coefficients, and p-values. Also presented are BLASTn matches of core OTUs to Bd associated taxa from Woodhams et al. 2015, with % identity and max bit scores. [file 42523_2020_53_MOESM1_ESM.docx]

Table S1 Results of co-association analysis between *Btk 72* and all core OTUs presented along with genus identifications, correlation coefficients (τ), and P-values. Also presented are BLASTn matches of core OTUs to *Bd* associated taxa from Woodhams et al. 2015, with % identity and max bit scores. Where our OTUs had ≥99% identity matches with 100% query coverage, we call a match (shaded cells).

| Tested Core OTU | Genus | Kendall τ | P-value | Match to Woodhams et al. 2015 | % Identity | Max Score |
| --- | --- | --- | --- | --- | --- | --- |
| OTU1 | *Bordetella* | 0.114 | 0.035 | Litoriaserrata-inhibitory_7 | 99% | 435 |
| OTU2 | *Nesterenkonia* | 0.098 | 0.070 | Atelopuslimosus-enhancing_2 | 98% | 425 |
| OTU3 | *Caldalkalibacillus* | 0.125 | 0.021 | Colostethuspanamensis-ns_6 | 89% | 331 |
| OTU5 | *Acinetobacter* | 0.120 | 0.027 | Craugastorcrassidigitus-inhibitory_181 | 100% | 453 |
| **OTU9** | ***Propionibacterium*** | **0.238** | **<0.001*** | Plethodoncinereus-ns_13 | 90% | 345 |
| OTU10 | *Pseudomonas* | 0.004 | 0.938 | Silverstoneiaflotator-inhibitory_51 | 100% | 453 |
| **OTU15** | ***Flavobacterium*** | **0.308** | **<0.001*** | Hemidactylumscutatum-ns_5 | 100% | 453 |
| OTU16 | *Delftia* | 0.065 | 0.232 | Ranamuscosa-inhibitory_35 | 100% | 453 |
| OTU17 | *Halomonas* | 0.137 | 0.011 | Colostethuspanamensis-ns_4 | 88% | 322 |
| OTU18 | Comamonadaceae_unclassified | 0.019 | 0.716 | Craugastorcrassidigitus-inhibitory_98 | 100% | 453 |
| OTU20 | *Acinetobacter* | 0.126 | 0.020 | Smiliscasordida-enhancing_9 | 100% | 453 |
| OTU21 | *Cronobacter* | 0.064 | 0.239 | Craugastorcrassidigitus-inhibitory_105 | 100% | 453 |
| OTU24 | *Nesterenkonia* | 0.080 | 0.138 | Atelopuslimosus-enhancing_2 | 97% | 419 |
| **OTU25** | ***Enhydrobacter*** | **0.208** | **<0.001*** | Bufotyphonius-inhibitory_17 | 88% | 320 |
| OTU26 | *Caldalkalibacillus* | 0.113 | 0.037 | Plethodoncinereus-inhibitory_10 | 91% | 349 |
| **OTU28** | ***Dermacoccus*** | **0.205** | **<0.001*** | Hemidactylumscutatum-ns_8 | 96% | 408 |
| OTU29 | *Bacillus* | 0.143 | 0.008 | Ranamuscosa-inhibitory_70 | 97% | 421 |
| **OTU31** | ***Micrococcus*** | **0.231** | **<0.001*** | Smiliscasila-inhibitory_3 | 100% | 453 |
| OTU41 | Comamonadaceae_unclassified | -0.001 | 0.989 | Silverstoneiaflotator-ns_1 | 98% | 430 |
| OTU49 | *Methylophilus* | 0.046 | 0.396 | Silverstoneiaflotator-inhibitory_41 | 92% | 367 |
| **OTU52** | ***Sphingomonas*** | **0.251** | **<0.001*** | Atelopuselegans-ns_3 | 98% | 435 |
| **OTU76** | ***Corynebacterium*** | **0.232** | **<0.001*** | Agalychniscallidryas-ns_25 | 91% | 351 |
| **OTU81** | ***Flavobacterium*** | **0.277** | **<0.001*** | Ranamuscosa-inhibitory_45 | 96% | 408 |
| OTU100 | *Geobacillus* | 0.097 | 0.074 | Plethodoncinereus-inhibitory_10 | 95% | 399 |
| OTU102 | *Caldalkalibacillus* | 0.038 | 0.479 | Plethodoncinereus-inhibitory_10 | 90% | 345 |
